# Renal Tubular Complement 3 Deposition in Children with Primary Nephrotic Syndrome

**DOI:** 10.1155/2018/4386438

**Published:** 2018-05-30

**Authors:** Fengying Wang, Xiaozhong Li, Xueming Zhu, Qing Chen, Lu Jiang, Ziqiang Zhu

**Affiliations:** ^1^Department of Nephrology, Children's Hospital of Soochow University, Suzhou, China; ^2^Department of Pediatrics, Taixing Hospital Affiliated to Yangzhou University, Taixing, Jiangsu, China; ^3^Department of Pathology, Children's Hospital of Soochow University, Suzhou, China

## Abstract

**Background:**

This study aimed to investigate the clinical significance of complement 3 (C3) deposition in renal tubules of children with primary nephrotic syndrome (PNS).

**Methods:**

The clinical and pathological characteristics of PNS were retrospectively reviewed in 99 PNS pediatric patients, who were divided into the C3 deposition and the non-C3 deposition groups.

**Results:**

A total of 39 patients (39.39%) had renal tubule C3 deposition. In the C3 deposition group, the ratios of urine N-acetylglucosaminidase/creatinine (UNAG/Cr), urine *β*2 microglobulin/creatinine (U*β*2MG/Cr), and urine transferrin/creatinine (UTRF/Cr) were significantly higher than those of the non-C3 deposition group. The patients of the C3 deposition group had lower serum total protein and albumin, higher cholesterol and D-dimer (DD), lower proportion of CD3+CD8+ cells, and higher proportion of CD19+CD23+ cells. The number of the patients with interstitial fibrosis, renal cell vacuolar degeneration, renal tubular immunoglobulin deposition, and severe tubulointerstitial injury in the C3 deposition group was higher than that of the non-C3 deposition group. The C3 deposition intensity was positively correlated with the number of recurrences.

**Conclusion:**

PNS pediatric patients with C3 deposition in renal tubules have more severe disease condition, tubulointerstitial injury, and recurrence suggesting a worse long-term prognosis.

## 1. Introduction

Primary nephrotic syndrome (PNS) is a common primary glomerular disease in children and accounts for more than 90% of nephrotic syndrome (NS) in childhood [1–3]. In recent years, the incidence of PNS has increased and the incidence of children with PNS that are resistant to initial steroid treatment is also increasing [2, 4]. PNS can be found in a variety of pathological types that are categorized according to the change in nephrosis and are classified as minimal change disease and nonminimal change disease. The immunoglobulin deposition and/or complement deposition are common biological processes in the kidney tissues of patients with nonminimal change disease, whereas they are not seen in the glomeruli of patients with minimal change disease, which may be related to T cell dysfunction.

Complement 3 (C3) and its active products in the kidneys have been studied in specific renal diseases, and the majority of the studies have focused on the role of glomerular immunoglobulin (IgA) deposition in IgA nephropathy (IgAN). There is evidence showing that glomerular C3 deposition is related to the activity, severity, and prognosis of IgAN [5–7]. Patients with primary focal segmental glomerulosclerosis (FSGS) and glomerular IgM and C3 deposition indicated a poorer response to pharmacotherapy and exhibited a poorer renal prognosis [8]. However, a limited number of studies have been conducted in order to investigate the renal tubular C3 deposition in subjects with renal diseases, whereas little is known regarding the clinical significance of C3 deposition, notably in pediatric patients with PNS. The majority of the studies that have examined the association between complement C3 levels and nephropathies have been conducted in adults [9, 10]. The studies that have addressed the impact of immunoglobulins and/or complement C3 deposits in young patients with glomerulonephritis were reported approximately 20 years ago [11, 12]. More recent studies that have focused on glomerulopathies and complement C3 deposition were isolated case reports or involved a small range of case reports and did not exhibit the characteristics of a retrospective and/or a cross-sectional clinical study analysis [13–15]. To the best of our knowledge no study has been conducted to address the clinical significance of renal tubular C3 deposition in pediatric patients with PNS. In the present study, the clinical and pathological characteristics of pediatric patients with PNS were retrospectively reviewed. The study aimed to explore the clinical significance of renal tubules C3 deposition in pediatric patients with PNS.

## 2. Materials and Methods

### 2.1. Patients

A total of 99 children with PNS who received renal biopsy between January 2010 and December 2016 were retrospectively reviewed. The inclusion criteria were as follows: the age of the patients was between 1 and 18 years and they were diagnosed with PNS according to the International Study of Kidney Disease in Children (ISKDC) diagnostic criteria for NS [16]. The exclusion criteria were according to the following: Secondary NS and patients with severe pediatric diseases were excluded. Severe pediatric diseases included diabetes, malignant tumors, aplastic anemia, severe myocarditis, acute or subacute severe hepatitis, primary or secondary immunodeficiency disease, and Epstein-Barr virus or cytomegalovirus infection.

On the basis of renal immunopathology whether the renal tubules had C3 deposition, patients were divided into the C3 deposition and the non-C3 deposition groups. The study was approved by the institutional review board and the research ethics committee of the Children's Hospital of Soochow University (number 2017LW001), and all clinical investigations were conducted according to the principles and guidelines provided by the Declaration of Helsinki. Written informed consent was acquired and patient records were anonymous prior to analysis.

### 2.2. Demographic Data and Laboratory Test

The demographic data included gender, age of renal biopsy, course of disease prior to the renal biopsy, initial diagnosis and/or frequency of recurrence, blood pressure, hematuria, urine protein quantification, urine protein spectrum, blood biochemical findings, results from humoral immunity examination, cellular immunity examination, and coagulation function examination. The urine protein analysis included N-acetylglucosaminidase (UNAG), *α*_1_ microglobulin (U*α*_1_MG), *β*_2_ microglobulin (U*β*_2_MG), IgG (UIgG), urinary creatinine (Cr), microalbumin (UMA), and transferrin (UTRF). The expression levels of these proteins were normalized according to Cr.

### 2.3. Urine Protein Profile

The morning urine was collected for the determination of the urinary protein profile by immunoturbidimetry. The assay was carried out using a commercial kit (Japan Union Pharmaceutical Co., Ltd.) in order to detect the NAG. A similar commercial ELISA kit (Shanghai LanYi Technology Co., Ltd.) was used for the measurement of *α*1MG and *β*2MG. The kits for the detection of IgG, MA, TRF, and Cr were purchased from Orion Corporation (Finland).

### 2.4. Lymphocyte Subgroups

A total of 3 ml of fasting peripheral venous blood from each patient was collected for analysis using a procoagulation tube on the same day and/or the next morning following admission. Patients with acute infectious or allergic diseases were excluded. The peripheral blood lymphocytes and their subgroups were determined using double-labeling method by flow cytometry (BD, USA). The CD3+, CD3+CD4+, and CD3+CD8+ cells were detected by a BD FACS Canto II flow cytometer. The CD3−CD19+ and CD3 − (CD16+56+) cells and CD19+CD23+ cells were measured by a BD FACS Canto II flow cytometer. The antibodies for flow cytometry were purchased from the BD Company (USA) and the procedures were performed according to the manufacturer's instructions.

### 2.5. Pathological Examination

The renal tissues that were collected following biopsy were subjected to routine light microscopy, immunofluorescence staining, and electron microscopy. The number of patients with renal pathology was recorded. The presence of severe mesangial cell proliferation, glomerular fibrous sclerosis, and/or crescent was suggestive of severe glomerular injury according to the association between pathological changes and poor renal prognosis and previously reported findings [17, 18]. The presence of tubular epithelial cell necrosis, tubular atrophy, interstitial inflammatory cell infiltration, and/or interstitial fibrosis was suggestive of severe tubulointerstitial injury [17, 18]. The number of patients with severe glomerular injury and/or severe tubulointerstitial injury was recorded.

### 2.6. Renal Biopsy

The patients were placed on a prone position. Both sides of the kidney area were raised. The local skin was disinfected. The 2% lidocaine was injected at the puncture point for anesthesia. Percutaneous renal puncture was conducted under the guidance of the ultrasound. The biopsy gun was stimulated when the needle reached the renal capsule. The needle was withdrawn immediately after the gunshot. Paraffin sections, frozen sections, and electron microscopy sections were produced from the samples of each patient. The tissues for paraffin sections were fixed with 10% formaldehyde, whereas the tissues for frozen sections were immediately cryopreserved for inspection. The tissues were examined by electron microscopy (JEM-1400/JEM-1400 PLUS, Japan) using 2.5% neutral glutaraldehyde.

Histological analysis included H&E, PAS, PASM, and Masson staining. The immunofluorescence assay included IgA, IgG, IgM, C3, C1q, and Fib. The semiquantitative method was used in order to evaluate the fluorescent deposition intensity: negative was denoted as “−”; visible at high magnification was denoted as “±”; visible at low magnification and apparently visible at high magnification were denoted as “+”; apparently visible at low magnification and clearly visible at high magnification were denoted as “++”; clearly visible at low magnification and dazzling at high magnification were denoted as “+++”; dazzling at low magnification and glaring at high magnification were denoted as “++++”.

### 2.7. Statistical Analysis

Statistical analysis was conducted using SPSS 19.0 software (SPSS, Chicago, IL, USA). Quantitative data with normal distribution were compared with the *t*-test, whereas those with abnormal distribution with the Wilcoxon rank sum test. The qualitative data were compared with the Chi-squared test (when total frequency [*n*] was ≥40 and each theoretical frequency [*T*] was ≥5), the Chi-squared test of continuity correction (when *n* was ≥40 and *T* was ≥1 but <5) and/or Fisher's exact test (*n* was <40, *T* was <1 or the *P* value of Chi-squared test was approximately equal to the value of *α*). The correlation analyses between kidney tubules with or without C3 deposition, C3 density, and specific biochemical indicators and recurrence were conducted by Spearman rank correlation. A *P* value lower than 0.05 (*P* < 0.05) was considered statistically significant.

## 3. Results

### 3.1. General Characteristics

**Figure 1:**
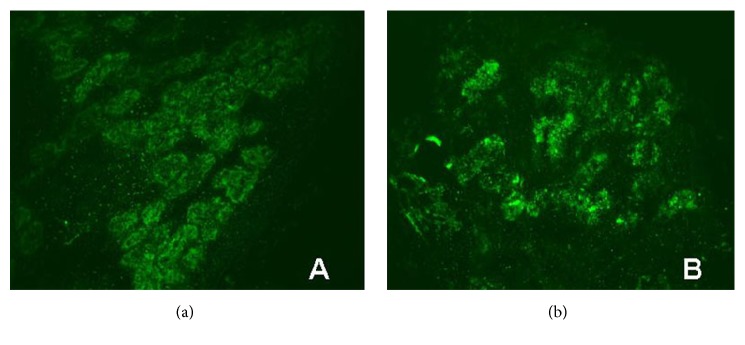
Immunofluorescence staining of C3 in renal tubular epithelial cells (×200). (a): C3 deposition in renal tubular epithelial cells (+). (b): C3 deposition in renal tubular epithelial cells (++).

A total of 99 children with PNS were included for analysis and among them 60 were male. Specifically, 39 cases (24 boys) were grouped according to the C3 deposition and 60 cases (36 boys) were grouped according to the non-C3 deposition. No significant differences were observed in the parameters gender, age of renal biopsy, course of disease prior to biopsy, incidences of macroscopic hematuria, microscopic hematuria, hypertension, and renal dysfunction between the groups (all *P* > 0.05) (Table 1 and Supplementary Table 1). The expression of C3 in the renal tubules is shown in Figure 1.

### 3.2. Urine Protein Quantification and Urine Protein Profile

No significant differences were noted in the 24 h urine protein (24U-TP) levels and the 24 h microalbumin concentration (24U-MA) between the two groups (*P* > 0.05). The UNAG/Cr, U*β*2MG/Cr, and UTRF/Cr in the C3 deposition group were significantly higher compared with those in the non-C3 deposition group (*P* < 0.05). Whereas no significant differences were observed with regard to the parameters U*α*1MG/Cr, UIgG/Cr, and UMA/Cr (*P* > 0.05) between the 2 groups (Table 1 and Supplementary Table 1.

### 3.3. Biochemical Results

In the C3 deposition group, the serum total protein and albumin were significantly lower than those in the non-C3 deposition group (*P* < 0.05). The serum cholesterol in the C3 deposition group was significantly higher than that noted in the non-C3 deposition group (*P* < 0.05). In addition, there were no significant differences in the parameters blood urea, creatinine, triglyceride, and Cys-C between the 2 groups (*P* > 0.05) (Table 1 and Supplementary Table 1).

### 3.4. Determination of Humoral Immunity, Complement, and D-Dimer (DD) Levels

The serum DD in the C3 deposition group was significantly higher than that in the non-C3 deposition group (*P* < 0.05). Moreover, no significant differences were noted with regard to the levels of IgA, IgG, IgM, C3, and C4 between the 2 groups (*P* > 0.05) (Table 1 and Supplementary Table 1).

### 3.5. Lymphocyte Measurement

**Figure 2:**
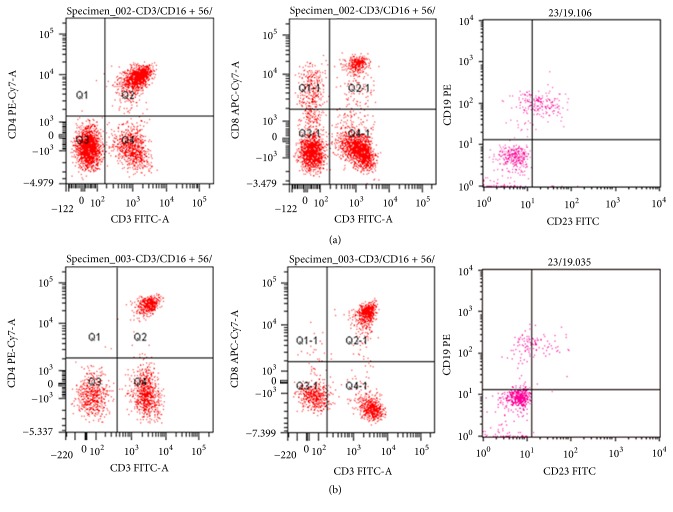
Representative images for CD3^+^CD4^+^, CD3^+^CD8^+^, and CD19^+^CD23^+^ of lymphocyte subpopulation. (a) The C3 deposition group: CD3^+^CD4^+^ : 33.5%, CD3^+^CD8^+^ : 11.6%, CD19^+^CD23^+^ : 21.67%; (b) the non-C3 deposition group: CD3^+^CD4^+^ : 26.7%, CD3^+^CD8^+^ : 36.8%, CD19^+^CD23^+^ : 7.63%.

The proportion of CD3+CD8+ cells in the C3 deposition group was lower compared with that in the non-C3 deposition group (*P* < 0.05). In the C3 deposition group, the proportion of CD19+CD23+ cells was significantly higher than that in the non-C3 deposition group (*P* < 0.05). There are no significant differences in the proportions of CD3+, CD3+CD4+, CD3−CD19+, and CD3−CD16+ CD56+ cells between the 2 groups (*P* > 0.05) (Table 1 and Supplementary Table 1). The representative figures for CD3+CD4+, CD3+CD8+, and CD19+CD23+ are shown in Figure 2.

### 3.6. Other Pathological Parameters

The data that were derived from light microscopy and immunofluorescence staining were collected from all the patients. In the C3 deposition group, the investigation using electron microscopy indicated no glomeruli in the 2 patients examined. Glomerular dissolution and degeneration was noted in 1 patient. In the non-C3 deposition group, the data derived by electron microscopy were missing in 8 patients, and thus the necessary information was available for 36 patients in the C3 deposition group and 52 patients in the non-C3 deposition group.

Severe tubulointerstitial injury was observed in 22 pediatric patients of the C3 deposition group and 14 pediatric patients of the non-C3 deposition group. A significant difference was noted between these 2 groups (*P* < 0.05; Table 1). The proportion of pediatric patients with severe glomerular injury in the C3 deposition group was slightly higher compared with that in the non-C3 deposition group (*P* > 0.05; Table 1). The proportion of pediatric subjects with interstitial fibrosis and/or vacuolar degeneration of renal tubular epithelial cells in the C3 deposition group was markedly higher than that in the non-C3 deposition group (*P* < 0.05). The majority of the patients exhibited glomerular IgM deposition, although no significant difference was noted with regard to the glomerular deposition among the patients that exhibited different levels of the parameters Igs, C3, C1q, and Fib (*P* > 0.05, Supplementary Table 2). In the C3 deposition group, the proportion of patients with fluorescence deposition of renal tubules was significantly higher than that in the non-C3 deposition group. Significant differences were noted in the proportions of patients with Ig deposition, IgG deposition, and IgG+IgA deposition between the 2 groups (*P* < 0.05, Supplementary Table 2). The data that were derived from the remaining electron and light microscopy observations were similar between the two groups with no apparent significant difference (*P* > 0.05) (Supplementary Table 2).

### 3.7. Correlation Analysis

The biochemical indicators that exhibited significant differences between the two groups and the indexes that revealed *P* values close to the statistical threshold of 0.05 were investigated by Spearman rank correlation analysis with the renal tubular C3 deposition. The results indicated a positive correlation between tubular C3 deposition and the parameters UNAG/Cr, U*β*2MG/Cr, UMA/Cr, UTRF/Cr, Cholesterol, IgM, DD, and CD4+/CD8+, respectively (Table 2, all *P* < 0.05). Furthermore, tubular C3 deposition negatively correlated with total protein, albumin, and CD3+CD8+ cells (Table 2, all *P* < 0.05), respectively. The correlation analysis further suggested that the intensity of the C3 deposition correlated positively with the number of recurrences (Table 2).

## 4. Discussion

The present study described the effects of the C3 deposition in tubular cells of pediatric patients with nephrotic syndrome. The data suggested that, in the C3 deposition group, the parameters that were associated with normal kidney function, such as urine N-acetylglucosaminidase/creatinine (UNAG/Cr) ratio, urine *β*2 microglobulin/creatinine (U*β*2MG/Cr) ratio, and urine transferrin/creatinine (UTRF/Cr) ratio, were significantly higher compared with those in the non-C3 deposition group. In addition, a lower serum total protein, albumin and proportion of CD3+CD8+ cells, and a higher proportion of CD19+CD23+ cells were noted in the C3 deposition group, whereas the subjects of this group revealed significant deterioration of the renal tissues with regard to interstitial fibrosis, renal tubular epithelial cells vacuolar degeneration, renal tubular immunoglobulin deposition, and severe tubulointerstitial injury compared with the patients of the non-C3 deposition group. The study demonstrated for the first time that pediatric patients with PNS and C3 deposition in renal tubules exhibit a poor long-term prognosis due to extensive local tubulointerstitial injury and recurrence compared with those without C3 deposition.

The activation of the complement pathway plays an important role in the renal injury of patients with kidney diseases. The C3 is the most abundant type compared to other complement proteins. Glomerular C3 deposition has been studied in several kidney diseases. Certain studies have shown that glomerular C3 deposition is associated with the disease activity and the severity of kidney injury in IgAN and lupus nephritis (LN) [19–22]. It has been documented that mesangial C3 deposition is positively correlated with mesangial cell proliferation, tubular atrophy, and interstitial fibrosis. Glomerular capillary C3d deposition may also serve as a new marker in the pathological diagnosis of MN at stage I, in patients who are treated with glucocorticoids. In recent years, various studies have explored the C3 deposition in glomerular capillaries and arterioles in association with IgAN and have revealed that peritubular capillary C3 deposition may be used as a predictor of a poor prognosis, while C3 deposition in the renal arterioles may be considered a marker of atherosclerosis [20, 22]. In addition, C3 deposition in the glomerular capsule and/or arterioles is related to the clinical outcome of IgAN [17]. In the current study, the clinical and pathological characteristics of PNS pediatric patients with and without renal C3 deposition in tubules were compared. Our results showed that C3 deposition was present in 39.39% of PNS subjects. No marked differences were noted with regard to the general clinical characteristics between the two groups.

Previous studies have indicated that low albumin in NS patients is related to renal injury and prognosis of kidney disease. In the present study, the serum total protein and albumin in the C3 deposition group were lower than those noted in the non-C3 deposition group, suggesting that the renal injury was more severe in patients with renal tubular C3 deposition. NS is usually accompanied by lipid metabolism disorders of different severities and hypercoagulable states. The hypercoagulable state is attributed to the anticoagulation/coagulation system imbalance and fibrinolysis abnormalities, whereas it further relates to the low serum albumin and increase in cholesterol [23, 24]. The production and increase of DD reflects the activation of coagulation and fibrinolysis and has been used as a specific marker of hypercoagulable state and secondary hyperfibrinolysis. The present study demonstrated that the serum cholesterol and DD in the C3 deposition group were higher compared with those in the non-C3 deposition group, suggesting that subjects with C3 deposition exhibited severe lipid metabolism disorders and hypercoagulable states and were more likely to develop renal microthrombosis. Recent findings revealed that the podocyte injury may cause severe hypercholesterolemia [25]. Furthermore, higher blood cholesterol (>10 mmol/L) is an independent risk factor of steroid dependence in NS pediatric patients [26]. Based on these studies, it may be assumed that pediatric subjects with renal tubular C3 deposition may be more susceptible to podocyte injury and will consequently develop steroid therapy dependence. These patients with renal tubular C3 deposition will be more susceptible to recurrence following reduction of the steroid dose, which is one of the causes of refractory treatment and poor prognosis. Correlation analysis also suggested that the C3 deposition intensity correlated positively with the number of recurrences. Moreover, correlation analysis indicated that C3 correlated positively with serum cholesterol and DD and negatively with total protein and albumin, suggesting that renal tubular C3 deposition may aggravate hypercholesterolemia, hypercoagulability, and hypoproteinemia. The data are in agreement with previous studies that have demonstrated that deficiency of C3 in low-density lipoprotein receptor-deficient (*Ldlr*^−/−^) mice has been shown to result in larger atherosclerotic lesions, with increased lipid deposition and impaired lesion development beyond the foam cell stage [27].

The serum total protein and the serum albumin levels in the C3 deposition group were markedly lower compared with the corresponding levels of these parameters in the non-C3 deposition group, although no significant differences in the 24U-TP and 24U-MA between the two groups were noted. 24U-TP and 24U-MA are used to evaluate the protein secretion in a specific day, despite the variability in the daily urine protein secretion between days. The urine protein profile is used to evaluate the renal tubular function, and the majority of the studies have focused on UNAG, U*β*2MG, and U*α*1MG. The increased levels of the ratios of UNAG, U*β*2MG, and U*α*1MG to urine creatinine reflect the renal tubular dysfunction and may serve as markers of tubulointerstitial injury at an early stage [28–30]. The data reported in the current study are in concordance with the aforementioned studies since patients with C3 deposition exhibited more severe tubulointerstitial injury, which was consistent with these biochemical indices. In addition, correlation analysis demonstrated a positive correlation between renal tubular C3 deposition and UNAG/Cr, U*β*2MG/Cr, UMA/Cr, and UTRF/Cr, suggesting that renal tubular C3 deposition could lead to tubulointerstitial injury and the reduction in the reabsorption of the urinary protein.

The pathogenesis of PNS may be attributed to an immunological etiology. The diversity of the experimental methods used in previous studies for the determination of immunological parameters in PNS has contributed to the controversy of the experimental findings among these studies [31]. Early studies indicated that the variables IgG and IgA were reduced, whereas IgM was increased in NS patients. In the present study, the two groups revealed no significant difference in serum immunoglobulin levels. In children, the pathogenesis of PNS is related to T lymphocyte dysfunction. Certain studies have shown that the population of CD8+ T and CD4+ T cells is increased in PNS patients [31, 32]. A recent research showed that INS patients had increased expression of TNF-*α* in CD4-lymphocytes and reduced expression of IFN-*γ* in CD8-lymphocytes [33]. B lymphocytes are also involved in the pathogenesis of PNS. The number of B lymphocytes increased in the active phase of the disease and was significantly higher compared with that noted in healthy subjects, as demonstrated by two recent studies [34, 35]. Rituximab (a B cell monoclonal antibody) has been used as a therapeutic drug in order to reduce the number of B lymphocytes in children with nephrotic syndrome [36, 37]. The data of the current report showed that the amount of lymphocyte subsets was significantly different between patients with and without C3 deposition. Significant differences were observed in cytotoxic T cells (CD3+CD8+ cells) and activated B cells (CD19+CD23+ cells) between the two groups. In the C3 deposition group, the number of activated B lymphocytes was higher, suggesting that C3 deposition may serve as an indication for the use of the B cell monoclonal antibody.

In addition, the number of subjects with interstitial fibrosis, vacuolar degeneration of renal tubular epithelial cells, severe renal tubulointerstitial injury, and renal tubular Ig deposition in C3 deposition group was significantly higher compared with that in the non-C3 deposition group. Thus, patients with C3 deposition exhibit tubulointerstitial lesions with greater severity. The severity of tubulointerstitial lesions is a key factor in determining the long-term kidney function of patients with glomerular disease. Notably, tubulointerstitial fibrosis and tubular atrophy are determinants of the progression of primary kidney disease [38, 39]. Thus, it is assumed that patients with renal tubular C3 deposition exhibit a poorer prognosis compared with those without renal tubular C3 deposition. The positive correlation between C3 deposition intensity and the recurrence times further suggests that subjects with C3 deposits have a worse prognosis. Renal tubular epithelial cells, glomerular epithelial cells, mesangial cells, and infiltrating cells may produce C3. In renal injury or the acute phase of inflammation, the levels of local C3 increase in the kidney. Under pathological conditions, this increase may facilitate the progression of kidney disease [40, 41]. In the current study, no significant differences were noted in the serum C3 levels and the glomerular C3 deposition number between the two groups of patients. In addition, no correlation between glomerular Ig fluorescence deposition and tubular C3 deposition was observed, although tubular Ig deposition and tubular C3 deposition correlated with each other. The C3 deposition of patients with interstitial fibrosis and severe tubulointerstitial injury was higher than that observed in the patients without C3 deposition. The data suggested that the main source of C3 tubular deposition in this group of subjects originated mainly from local synthesis of tubules, whereas tubular deposition of C3 participated in subjects with tubulointerstitial injury. A previous study has shown that C3 deposition in renal tubules is related not only to interstitial inflammation but also directly to the progression of interstitial fibrosis [42]. In the current study, the number of patients with interstitial inflammatory cell infiltration in the C3 deposition group was only slightly higher than that in the non-C3 deposition group, which might be due to the small sample size of the population examined. Gherghiceanu et al. [20] suggested that glomerular C3 deposition was related to the active IgAN, and C3 deposition in renal tubular epithelial cells and peritubular capillaries were associated with sclerosis, suggesting that the pattern of C3 deposition may be used to predict the progression of nephropathy and the deterioration of kidney function. Our findings support the aforementioned findings and suggest that C3 deposition in renal tubules is related to the interstitial fibrosis and sclerosis and can be used as a prognostic marker for renal tubular cell injury.

To the best of our knowledge no study has examined the effect of renal tubular C3 deposition in pediatric subjects with nephrotic syndrome with regard to the number of T lymphocytes and the biochemical parameters albumin, cholesterol, and D-dimer levels (DD). A previous study examined the effect of the deposition of C3a and C5a in 83 patients with renal biopsy proven IgA nephropathy [6] and concluded that the deposition increased with increasing grades of renal pathology in IgAN patients, while it correlated with proteinuria and serum creatinine (SCr), which is in agreement with our findings.

This study is not without limitations. Firstly, the fluorescence intensity of C3 was not evaluated in different patients, since the number of the subjects in a specific group was very small. Secondly, the association of the C3 deposition with the response to steroid treatment was not further assessed due to some objective reasons such as the missing visits of certain patients and the time limit.

## 5. Conclusions

Taken together, the results indicate that PNS in pediatric patients with C3 deposition in renal tubules exhibits severe renal injury and poorer prognosis compared with pediatric patients without C3 deposition. It is suggested that renoprotective treatment should be initiated for PNS pediatric patients following the detection of renal tubular C3 deposition in order to delay the deterioration of kidney function.

## Figures and Tables

**Table 1:** Clinical and pathological parameters between C3 deposition and non-C3 deposition groups.

Items	C3 deposition group (*n* = 39)	Non-C3 deposition group (*n* = 60)	*P*
Gender (males)/*n*	24	36	0.878
Age at biopsy (yr) ($\overset{-}{x}\pm s$)	7.58 ± 3.97	8.40 ± 3.41	0.275
24U-TP/kg ($\overset{-}{x}\pm s$)	144.68 ± 77.84	119.12 ± 72.37	0.141
24U-MA/kg ($\overset{-}{x}\pm s$)	35.07 ± 22.88	32.08 ± 23.37	0.577
UNAG/Cr (M, Q)	6.78, 6.43	4.66, 3.87	0.004
U*β*2MG/Cr (M, Q)	0.13, 0.12	0.06, 0.12	0.009
UTRF/Cr ($\overset{-}{x}\pm s$)	40.99 ± 29.73	24.81 ± 20.80	0.002
Total protein (g/L)	44.14 ± 5.72	50.66 ± 9.66	<0.001
Albumin (g/L)	22.07 ± 5.56	27.13 ± 8.38	0.001
Cholesterol (mmol/L)	10.07 ± 3.67	8.15 ± 2.86	0.005
Triglycerides (mmol/L)	2.58 ± 1.34	2.25 ± 1.24	0.217
DD (*μ*g/L)	874.61 ± 661.77	436.48 ± 558.51	0.002
CD3+CD4+	34.01 ± 6.79	33.40 ± 7.79	0.708
CD3+CD8+	27.58 ± 5.38	30.89 ± 6.67	0.016
CD4+/CD8+	1.28 ± 0.37	1.13 ± 0.42	0.095
CD19+CD23+	9.39 ± 5.98	6.92 ± 3.17	0.014
Severe glomerular injury/*n* (%)	19 (48.72)	25 (41.67)	0.490
Severe tubulointerstitial injury/*n* (%)	22 (56.41)	14 (23.33)	0.001

**Table 2:** Correlation analysis.

	*r*_*s*_	*P*
UNAG/Cr	0.30	0.003
U*α*1MG/Cr	0.19	0.066
U*β*2MG/Cr	0.30	0.003
UIgG/Cr	0.17	0.097
UMA/Cr	0.21	0.042
UTRF/Cr	0.35	<0.001
Total protein	−0.36	<0.001
Albumin	−0.29	0.004
Cholesterol	0.25	0.013
IgG	−0.19	0.068
IgM	0.24	0.021
DD	0.39	0.001
CD3+CD8+	−0.27	0.012
CD4+/CD8+	0.22	0.042
CD19+CD23+	0.12	0.262
Recurrence times	0.45	0.010

## Data Availability

No additional data are available.

## Supplementary Materials

Supplementary Materials Supplementary Table 1: additional clinical and pathological parameters between C3 deposition and non-C3 deposition groups. Supplementary Table 2: additional pathological parameters between C3 deposition and non-C3 deposition groups.
