# Supplementary material for: Renal Tubular Complement 3 Deposition in Children with Primary Nephrotic Syndrome
Source: Biomed Res Int. 2018 May 30;2018:4386438. doi: 10.1155/2018/4386438 (PMC5998187; doi:10.1155/2018/4386438)
Supplement: Supplementary Materials — Supplementary Table 1: additional clinical and pathological parameters between C3 deposition and non-C3 deposition groups. Supplementary Table 2: additional pathological parameters between C3 deposition and non-C3 deposition groups. [file 4386438.f1.pdf]

**Supplementary table 1** Additional clinical and pathological parameters between C3 deposition and non-C3 deposition groups

| Items | **C3 deposition group (n=39)** | **non-C3 deposition group (n=60)** | **P** |
|---|---|---|---|
| Course of disease before biopsy (days ) (M,Q) | 28, 330 | 77.5, 427 | 0.103 |
| Macroscopic hematuria (n) | 4 | 6 | 1.000 |
| Microscopic hematuria (n) | 19 | 26 | 0.599 |
| Hypertension (n) | 6 | 8 | 0.775 |
| Renal dysfunction (n) | 3 | 1 | 0.334 |
| Uα1MG/Cr (M,Q) | 4.08, 3.91 | 3.17, 3.23 | 0.083 |
| UIgG/Cr (M,Q) | 20.59, 23.62 | 14.13, 26.52 | 0.072 |
| UMA/Cr ($\bar{x} \pm s$) | 131.01±121.36 | 91.98±97.65 | 0.082 |
| creatinine (μmol/L) | 50.03±31.13 | 46.71±27.25 | 0.581 |
| Urea (mmol/L) | 6.51±6.10 | 5.54±3.87 | 0.338 |
| Cys-C (mg/L) | 0.82±0.31 | 0.89±0.51 | 0.555 |
| IgA ( g/L) | 1.53±0.65 | 1.57±0.71 | 0.776 |
| IgG ( g/L) | 3.94±1.91 | 4.82±2.71 | 0.090 |
| IgM ( g/L) | 1.86±0.65 | 1.60±0.61 | 0.052 |
| C3 ( g/L) | 1.07±0.25 | 1.12±0.33 | 0.442 |
| C4 ( g/L) | 0.30±0.10 | 0.30±0.11 | 0.953 |
| CD3+ | 66.67±8.37 | 69.49±8.16 | 0.121 |

| | | | |
|---|---|---|---|
| CD3-CD19+ | 18.48±8.75 | 17.40±6.86 | 0.523 |
| CD3-CD(16+56) | 12.91±7.78 | 10.92±6.75 | 0.207 |

**Supplementary table 2** Additional pathological parameters between C3 deposition and non-C3 deposition groups

| Items | C3 deposition group (n=39) | non-C3 deposition (n=60) | P |
|---|---|---|---|
| Microscopy/n (%) | | | |
| Moderate to severe mesangial hyperplasia | 5(12.8) | 3(5) | 0.309 |
| severe mesangial hyperplasia | 1(2.56) | 3(5) | 0.937 |
| Mesangial hyperplasia with focal segmental aggravation | 3(7.69) | 7(11.67) | 0.764 |
| Glomerular sclerosis | 1(2.56) | 2(3.33) | 1.000 |
| Glomerular fibrosis, waste glomeruli | 6(15.38) | 5(8.33) | 0.445 |
| Crescent | 0(0) | 2(3.33) | 0.518 |
| Capsular adhesion and peribulbar fibrosis | 3(7.69) | 9(15) | 0.439 |
| Capillary collapse, occlusion | 11(28.21) | 13(21.67) | 0.458 |
| RBC cast in renal tubules | 5(12.8) | 9(15) | 0.761 |
| Swelling of renal tubular epithelial cells | 21(53.85) | 29(48.33) | 0.592 |
| Necrosis and atrophy of renal tubular epithelial cell | 2(5.13) | 1(1.67) | 0.703 |
| Interstitial inflammatory cell infiltration | 13(33.33) | 10(16.67) | 0.087 |
| Interstitial fibrosis | 7(17.95) | 3(5) | 0.046 |
| Small vessel disease | 0(0) | 1(1.67) | 1.000 |

| | | | |
|---|---|---|---|
| Immunofluorescence staining/n (%) | | | |
| Glomerular fluorescence deposition | | | |
| IgG | 0(0) | 2(3.33) | 0.518 |
| IgA | 4(10.25) | 1(1.67) | 0.151 |
| IgM | 15(38.46) | 22(36.67) | 0.857 |
| IgA+IgM | 6 (15.38) | 13(21.67) | 0.438 |
| IgG+IgM | 0(0) | 4(6.67) | |
| IgG+IgA+IgM | 3(7.69) | 8(13.33) | 0.585 |
| C3 | 6(15.38) | 14(23.33) | 0.336 |
| C1q | 5(12.82) | 11(18.33) | 0.467 |
| Fib | 13(33.33) | 28(46.67) | 0.188 |
| No fluorescence | 10（25.64） | 8（13.33） | 0.121 |
| Renal tubular fluorescence deposition | | | |
| IgG | 13(33.33) | 3(5) | 0.000 |
| IgG+IgA | 8(20.51) | 0(0) | 0.001 |
| IgG+IgA+IgM | 2(5.13) | 0(0) | 0.153 |
| Ig deposition (total n) | 23(58.97) | 3(5) | 0.000 |
| Fib | 3(7.69) | 0(0) | 0.114 |
| Electric microscopy /n(%) ( effective: n=36 and n=52) | | | |
| Basement membrane thickening | 4(11.11) | 11(21.15) | 0.218 |
| Uneven basement membrane | 3(8.33) | 2(3.85) | 0.670 |

| | | | |
|---|---|---|---|
| Podocytic process fusion | 31(86.11) | 38(73.08) | 0.144 |
| Podocytic process microvilli | 16(44.44) | 25(48.08) | 0.737 |
| Vacuolar degeneration and swelling of capillary endothelial cells | 11(30.56) | 10(19.23) | 0.220 |
| Capillary obstruction | 13(36.11) | 20(38.46) | 0.823 |
| Basement sediment | 6(16.67) | 10(19.23) | 0.759 |
| Vacuolar degeneration of renal tubular epithelial cells | 26(72.22) | 22(42.31) | 0.006 |
| Shedding of renal tubular epithelial cells | 3(8.33) | 3(5.77) | 0.969 |
